# Supplementary material for: Preferences for on-demand/intermittent/event-driven and long-acting injectable (LAI) HIV pre-exposure prophylaxis (PrEP) among HIV-negative Black gay, bisexual, and other sexual minority men in the United States: A qualitative study
Source: PLoS One. 2025 Jun 30;20(6):e0323212. doi: 10.1371/journal.pone.0323212 (PMC12208431; doi:10.1371/journal.pone.0323212)
Supplement: Attachment — Submitted filename: Response to Reviewer Comments.docx [file pone.0323212.s003.docx]

February 26, 2025

Emily Chenette, PhD

Editor-in-Chief

PLOS ONE

Dear Dr. Chenette,

We are pleased with the opportunity to resubmit our manuscript, *Preferences for on-demand/intermittent/event-driven and long-acting injectable HIV pre-exposure prophylaxis (PrEP) among HIV-negative Black gay, bisexual, and other sexual minority men in the United States: A qualitative study, in* consideration for publication as a research article in *PLOS ONE.* We believe we have addressed all points from the prior review and that the manuscript, in its’ current state is ready for publication.

We appreciated the thoughtful comments of the reviewers from our initial submission

On the page that follow, we include a table that outlines the reviewer comments and our corresponding responses. In the revised manuscript, changes are highlighted.

Please do not hesitate to contact me if you have any questions or require further information. Thank you for your consideration and we look forward to your response.

**Editors Comments**

| **Comments** | **Response** |
| --- | --- |
| Please ensure that your manuscript meets PLOS ONE's style requirements, including those for file naming | This has now been completed. |
| Please amend either the title on the online submission form (via Edit Submission) or the title in the manuscript so that they are identical. | This has now been completed. |
| Please amend your list of authors on the manuscript to ensure that each author is linked to an affiliation. Authors’ affiliations should reflect the institution where the work was done (if authors moved subsequently, you can also list the new affiliation stating “current affiliation:….” as necessary). | This has now been completed. |
| Please match your authorship list in your manuscript file and in the system. | This has now been completed. |
| PLOS requires an ORCID iD for the corresponding author in Editorial Manager on papers submitted after December 6th, 2016. Please ensure that you have an ORCID iD and that it is validated in Editorial Manager. To do this, go to ‘Update my Information’ (in the upper left-hand corner of the main menu), and click on the Fetch/Validate link next to the ORCID field. This will take you to the ORCID site and allow you to create a new iD or authenticate a pre-existing iD in Editorial Manager. | The ORCID ID has now been added to Editorial Manager |
| We note that the grant information you provided in the ‘Funding Information’ and ‘Financial Disclosure’ sections do not match. | The funding information are now congruent. |
| Thank you for stating the following financial disclosure:     [This study was funded by the National Institutes of Health (R34DA054870). Dr. Ogunbajo acknowledges salary support from U.S. National Institutes of Health K01MH129165.].    Please state what role the funders took in the study.  If the funders had no role, please state: ""The funders had no role in study design, data collection and analysis, decision to publish, or preparation of the manuscript.""    If this statement is not correct you must amend it as needed.    Please include this amended Role of Funder statement in your cover letter; we will change the online submission form on your behalf. | This has now been amended. |
| We note that you have indicated that there are restrictions to data sharing for this study. PLOS only allows data to be available upon request if there are legal or ethical restrictions on sharing data publicly. For more information on unacceptable data access restrictions, please see <http://journals.plos.org/plosone/s/data-availability#loc-unacceptable-data-access-restrictions>.    Before we proceed with your manuscript, please address the following prompts:    a) If there are ethical or legal restrictions on sharing a de-identified data set, please explain them in detail (e.g., data contain potentially identifying or sensitive patient information, data are owned by a third-party organization, etc.) and who has imposed them (e.g., a Research Ethics Committee or Institutional Review Board, etc.). Please also provide contact information for a data access committee, ethics committee, or other institutional body to which data requests may be sent.    b) If there are no restrictions, please upload the minimal anonymized data set necessary to replicate your study findings to a stable, public repository and provide us with the relevant URLs, DOIs, or accession numbers. For a list of recommended repositories, please see    <https://journals.plos.org/plosone/s/recommended-repositories>. You also have the option of uploading the data as Supporting Information files, but we would recommend depositing data directly to a data repository if possible.    We will update your Data Availability statement on your behalf to reflect the information you provide. | The data is now publicly available at:  [https://doi.org/10.5064/F6AW0DHShttps://doi.org/10.5064/F6AW0DHS](https://doi.org/10.5064/F6AW0DHS) |
| Please review your reference list to ensure that it is complete and correct. If you have cited papers that have been retracted, please include the rationale for doing so in the manuscript text, or remove these references and replace them with relevant current references. Any changes to the reference list should be mentioned in the rebuttal letter that accompanies your revised manuscript. If you need to cite a retracted article, indicate the article’s retracted status in the References list and also include a citation and full reference for the retraction notice. | This has been completed. |

**Reviewer #1 Comments**

| **Comments** | **Response** |
| --- | --- |
| It appears that each focus group included people who were on-PrEP, PrEP-naive, and who had discontinued PrEP. How could these different groups have influenced or biased each other in their responses during the focus group? Was this controlled for in any way? | We conducted focus group with three different groups: people who were on-PrEP, PrEP-naive, and who had discontinued PrEP. However, |
| A more detailed description of the qualitative data analysis is warranted. How many coders were there? How was interrater reliability assessed? As many items as you can complete from the COREQ (<https://www.hsph.harvard.edu/wp-content/uploads/sites/2448/2021/02/Consolidated-criteria-for-reporting-qualitative-research-COREQ.pdf>) would be informative. | The COREQ checklist has now been completed and included as a supporting document for the revision submission. |
| Was any other data gathered to assess participants' risk behaviors and how/whether they varied? Or the reasons why some of them had discontinued or decided not to get on PrEP? These could be informative contexts for their preferences around PrEP. | Unfortunately, we did not gather any additional data beyond brief demographic descriptive data to characterize the study sample. |

**Reviewer #2 Comments**

| **Comments** | **Response** |
| --- | --- |
| The authors present this as a study of Black gay, bisexual, and other sexual minority men. However, their eligibility criteria referred to sex assigned at birth—not gender—and not all participants identified as cisgender men. Additionally, the eligibility criteria made no mention of sexual orientation, and only 69% of participants identified as gay/homosexual. It is unclear from the Results section how the other 31% of participants identified (i.e., queer, bisexual/pansexual, straight/heterosexual, etc.), but at least one participant is described as “straight,” suggesting that not all participants identified as men who have sex with men. Furthermore, although the eligibility criteria for the study included identifying as African American/Black, 2% of participants in the sample did not identify as Black. The researchers should provide a demographics table that clearly describes the gender, sexual orientation, and race/ethnicity of the participants in the sample, and should not describe the sample as “58 HIV-negative Black SMM” if this does not accurately reflect the identities of all participants. | We have now provided a demographic table that clearly displays the gender, sexual orientation, and race/ethnicity of the participants in the sample. We have also edited the participant demographics section of the results to accurately reflect that. |
| Relatedly, it is commendable that the study sample included current PrEP users, PrEP non-users, and PrEP discontinuers, but it would be helpful if a demographics table noted how many participants fell into each category. Additionally, the authors seem to use the phrases “non-PrEP users” and “PrEP Naive Group” interchangeably, but these are not synonymous; one describes participants’ lack of experience taking PrEP, while the other describes participants’ lack of knowledge of PrEP. If all PrEP non-users in the sample were also PrEP naïve, this should be specified within the manuscript. | We have now included this information within the newly added demographics table. Our definition of PrEP naïve is related to having not ever utilized PrEP in the past not related to not being knowledgeable about PrEP. We recognize that is a clinical term and might be misinterpreted by different readers. To address this, We have now made that removed the term “naïve” and utilized “current non-PrEP users” instead. |
| The authors make a strong argument for focusing on Black SMM (because they are disproportionately affected by HIV, and less likely than their white counterparts to receive PrEP prescriptions). Although the introduction references structural inequities that contribute to these health disparities (e.g., racism), the authors may wish to expand their discussion of these systemic/structural factors, and emphasize them over patient-level factors (e.g., likelihood of following providers’ recommendations) that may be seen as blaming patients. Additionally, I would advise the authors to move away from "risk" language throughout the paper, as it can be stigmatizing. | We agree with these sentiments have now edited the manuscript accordingly. All risk related language has now been removed from the manuscript. |
| The authors organized the findings into two themes, which they defined as “lack of interest in on-demand PrEP” and “high acceptability of LAI-PrEP.” They then identify numerous “reasons” or “common sentiments” expressed by participants. The Results section would benefit from a reorganization that presents these common sentiments as the themes of interest, or as sub-themes under the previously identified umbrella categories. It would also be useful to provide more specific information about the number of participants who expressed each sentiment. | We agree with the reviewer and have now revised the results section accordingly. While we have now quantified, at the focus group discussion level, the level of acceptability of on-demand and LAI PrEP, we did not do so for each individual sentiment as we believe they are valid reasonings and explanations/support for the higher level general sentiments and attitudes towards on-demand and LAI PrEP and the level of cadence of the themes across FGDs does not take away from their importance. Additionally, as qualitative researcher, we push back against the sentiments that qualitative research findings have been to quantitatively be quantified to be believed as sound, empirical evidence. |
| According to the Methods section, the focus groups explored “PrEP initiation and adherence, social network composition and influence on PrEP use or lack thereof, and feedback on a planned social network strategy intervention to increase PrEP initiation and adherence among Black SMM.” It is unclear why analyses then exclusively focus on attitudes towards on-demand and LAI-PrEP. Specifically, why did the researchers not discuss attitudes towards daily dosing? Given that daily dosing was mentioned by multiple participants and is the only FDA-approved PrEP schedule, the omission was confusing. | These specific analyses focused on the attitudes towards on-demand and LAI-PrEP as that was the major focal point of this manuscript and we believe it worthy of a manuscript all by itself given the uniqueness of the dataset (including various different PrEP profiles). We did not explore attitudes towards daily dosing as that is the conventional typology of PrEP use and the vast majority of empirical research on PrEP is on daily-oral PrEP. |
| The authors provide very interesting illustrative quotations throughout the manuscript, but do not always integrate the quotations into the surrounding text. They may consider including fewer examples within the body of the text, and referencing a table with additional illustrative quotations. | We have now edited the in-text quotation but believe it is important to leave the qualitative data in-text rather than presenting it in a table as it allows for better ease of reading for the article reader. |
